# Neuromuscular Junction Degeneration in Skeletal Muscle Atrophy: Disease-Specific Mechanisms, Evidence-Informed Stratification, and Therapeutic Windows

**DOI:** 10.3390/ijms27209264

**Published:** 2026-10-19

**Authors:** Shiming Li, Mengyu Liu, Peng Zhang, Xuemei Ma, Xiaoping Chen

**Affiliations:** 1 College of Chemistry and Life Sciences, Beijing University of Technology, Beijing 100124, China; 15651746396@emails.bjut.edu.cn (S.L.); mengyu@bjut.edu.cn (M.L.); 2 National Key Laboratory of Human Factors Engineering, China Astronaut Research and Training Center, Beijing 100094, China; 3 National Key Laboratory of Space Medicine, China Astronaut Research and Training Center, Beijing 100094, China; zhangpeng6128@163.com

**Keywords:** neuromuscular junction, skeletal muscle atrophy, agrin-MuSK signaling, evidence stratification, translational biomarkers, therapeutic window

## Abstract

Skeletal muscle atrophy involves not only the loss of muscle mass and fiber size, but also a significant decline in contractile performance. Force decline often occurs before visible fiber atrophy, suggesting that early defects in neuromuscular transmission might trigger or exacerbate the wasting process. Therefore, we argue that neuromuscular junction (NMJ) degeneration is not a universal, conserved pathway across all atrophy syndromes. Instead, it should be viewed as a stage- and disease-specific event, with its actual contribution varying based on specific evidence. To explore this, we compare NMJ maintenance and degeneration across a broad spectrum of conditions, ranging from primary neuromuscular diseases (like denervation and motor neuron disorders) to systemic states such as aging, cachexia, and metabolic or respiratory diseases. Based on this, we propose a framework to evaluate NMJ involvement by timing, lesion type, and reversibility, drawing on diverse evidence from morphological, electrophysiological, and biomarker studies. Crucially, this approach distinguishes primary synaptic disorders from systemic conditions where NMJ damage acts merely as a secondary modifier. Ultimately, aligning specific molecular lesions with practical intervention windows will help refine patient stratification and guide the design of more targeted NMJ therapies.

## 1. Introduction

Skeletal muscle atrophy accompanies diverse clinical conditions, including aging, physical disuse, peripheral nerve injury, neurodegenerative disease, systemic inflammation, metabolic dysfunction, cancer cachexia, and treatment-related toxicity. Although their etiologies differ, these conditions converge on reduced myofiber dimensions, impaired force generation, and functional decline. The classic myofiber-centered paradigm, which emphasizes an imbalance between protein synthesis and degradation through ubiquitin-proteasome, autophagy-lysosome, and IGF-1-Akt pathways, remains foundational [1]. However, this paradigm does not fully explain why muscle weakness often precedes measurable fiber atrophy (dynapenia), or why restoration of muscle mass does not consistently restore contractile force. These discrepancies indicate that the rate-limiting lesion responsible for early functional impairment may lie upstream of the contractile apparatus.

Impaired neuromuscular transmission is a major candidate mechanism for this early functional decline. The neuromuscular junction (NMJ), the specialized chemical synapse linking motor neurons to skeletal myofibers, maintains excitation-contraction coupling through a high transmission safety factor [2]. Erosion of this safety margin can reduce force output before overt histological atrophy is apparent. Evidence from sarcopenia, disuse, denervation, amyotrophic lateral sclerosis (ALS), congenital myasthenic syndromes (CMS), and systemic disorders indicates that NMJ remodeling is not merely a late consequence of muscle loss [3,4]. Nonetheless, the strength and directness of this evidence vary substantially. In ALS, distal synaptic dismantling precedes motoneuron soma loss, consistent with a classical “dying-back” axonopathy [5]. In aging and disuse, transmission failure and endplate fragmentation often correlate more closely with functional weakness than with muscle mass alone. By contrast, in cancer cachexia, diabetes, chemotherapy toxicity, and chronic obstructive pulmonary disease (COPD), NMJ alterations are currently better interpreted as secondary modifiers or associated lesions rather than established primary drivers.

Structural and functional stability of the mature NMJ depends on continuous coordination among several homeostatic modules. The agrin-LRP4-MuSK signaling axis regulates postsynaptic acetylcholine receptor (AChR) clustering, density, and metabolic stability [6]. Presynaptic and subsynaptic mitochondrial networks provide local ATP for vesicle recycling and buffer intracellular calcium flux. Autophagy and mitophagy remove damaged macromolecular complexes and dysfunctional organelles [7], whereas perisynaptic Schwann cells survey synaptic integrity, clear debris, and provide neurotrophic support [8]. Failure of any one module can weaken neuromuscular transmission, but the initiating trigger and temporal sequence of degeneration differ markedly among clinical entities. Treating NMJ degeneration as a uniform pathway risks obscuring rate-limiting targets and misjudging the time window in which biological rescue remains feasible.

Much of the existing literature emphasizes individual diseases or single signaling pathways, which makes it difficult to determine whether NMJ degeneration functions as a primary driver, a secondary modifier, or a bidirectional partner in different muscle-wasting conditions. To address this limitation, peer-reviewed studies published between 2000 and 2026 in PubMed, Web of Science, and Scopus were synthesized into an evidence-informed framework that stratifies NMJ pathology across multiple qualitative domains (Table 1). Representative citation anchors and principal limitations underlying each evidence rating in Table 1 are summarized in Supplementary Table S1. The review first summarizes baseline mechanisms of NMJ homeostasis, then maps disease-specific degenerative patterns, evaluates diagnostic biomarkers, and discusses mechanism-guided therapeutic strategies alongside realistic intervention windows. Genetically defined myasthenic disorders and dystrophinopathies are used as mechanistic comparators to illustrate how primary synaptic or postsynaptic instability can shape weakness and wasting. The analysis is organized around three central questions: when neuromuscular transmission fails, which molecular lesion is rate-limiting, and to what extent the degenerative changes remain structurally or functionally reversible.

## 2. Normal Structure and Homeostatic Maintenance Mechanisms of the Neuromuscular Junction

### 2.1. Anatomical Structure and Cellular Components

The NMJ comprises a presynaptic motor terminal, a synaptic cleft, and a postsynaptic motor endplate. Motor axons branch into boutons enriched with acetylcholine-containing vesicles. Active-zone proteins, including ELKS/CAST family members, RIM, Munc13, and P/Q-type calcium channels (Cav2.1), couple action potential arrival to rapid calcium-dependent vesicle fusion [9,10].

The 50–80 nm synaptic cleft contains basal lamina components and acetylcholinesterase (AChE), which terminates acetylcholine signaling [11]. Postsynaptic junctional folds concentrate AChRs at their crests and Nav1.4 channels within the troughs, supporting efficient depolarization. Perisynaptic Schwann cells cover the terminal and actively participate in maintenance and repair through debris clearance, modulation of transmission, and release of trophic factors such as GDNF and TGF-beta [12].

### 2.2. Molecular Cascade and Homeostatic Regulation of the Agrin-MuSK Signaling Axis

The agrin-LRP4-MuSK cascade is required for NMJ formation and adult synaptic maintenance. Neural agrin, especially the z+ splice form released by motor neurons, binds LRP4 on the postsynaptic membrane through its C-terminal laminin G-like domains. This interaction promotes assembly and activation of the LRP4-MuSK complex and induces MuSK trans-phosphorylation on intracellular tyrosine residues [13]. The magnitude and duration of MuSK activity determine the stability of postsynaptic AChR clusters.

Activated MuSK recruits Dok-7, Crk/Crk-L, Src-family kinases, and rapsyn, thereby stabilizing AChR clusters [14]. Mutations in *AGRN*, *LRP4*, *MUSK*, *DOK7*, or *RAPSN* cause CMS or severe developmental synaptic defects [15]. Negative regulators, including Shp2, PTP1B, and Cbl-family ubiquitin ligases, counterbalance this pathway [16,17]. Recent work suggests that multivalent interactions among rapsyn, AChR-associated proteins, and scaffolds may involve phase-separation-like behavior, although its in vivo relevance remains unresolved. Because adult endplates require continuous AChR renewal, disruption of signal amplification or turnover control can destabilize the synapse under stress.

### 2.3. Synergistic Homeostatic Regulation of the Autophagy-Lysosome System and Mitochondrial Dynamics

Quality control of proteins and organelles contributes to NMJ maintenance. Deletion of the autophagy gene *Atg7* specifically in muscle results in aberrant AChR turnover, fragmentation of the postsynaptic apparatus and premature aging-like NMJ degeneration [7]. The E3 ligase MuRF1 recognizes AChRs once they have been released from rapsyn and promotes their ubiquitin-dependent degradation. K48-linked chains lead to proteasomal turnover while K63-linked chains can recruit p62/SQSTM1 leading to substrate targeting towards autophagic degradation [18]. Similarly, mitophagy plays an essential role at the presynaptic terminal: failure of PINK1-Parkin-mediated clearing of depolarized mitochondria induces accumulation of damaged organelles, altered calcium handling and terminal degeneration [19].

Mitochondrial biology represents a vulnerability module but not isolated proof of disease-specific NMJ causality. Terminal and subsynaptic mitochondria supply ATP required for acetylcholine synthesis, vesicle cycling, and calcium buffering; disruption of mitophagy, disturbance of NCLX-dependent calcium handling and disrupted ER-mitochondria contacts can impair synaptic transmission in experimental systems [19,20,21,22]. These pathways are particularly instructive in atrophy contexts when analyzed alongside structural or electrophysiological NMJ readouts.

**Figure 1:**
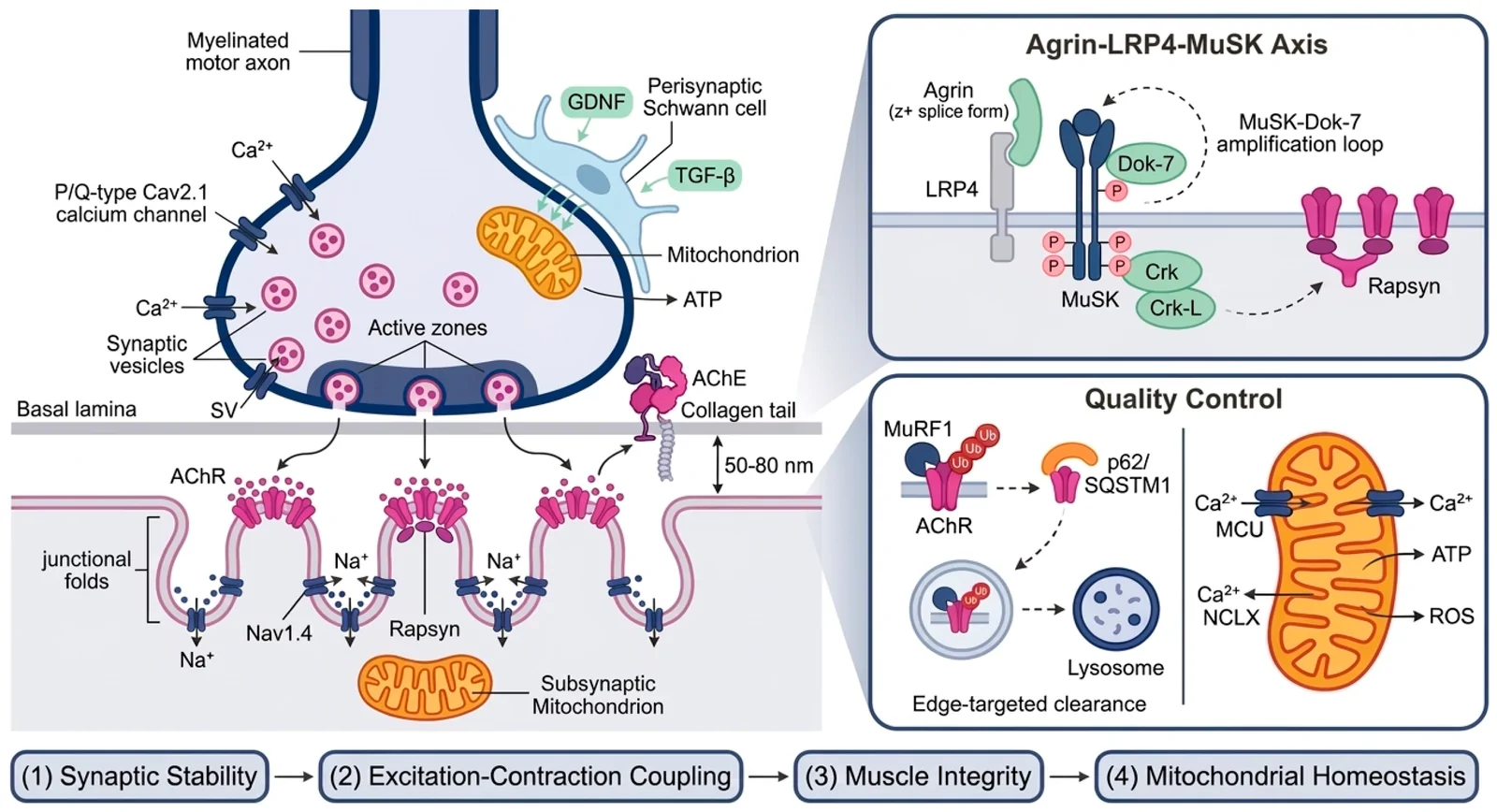
Molecular architecture and homeostatic maintenance of the neuromuscular junction. Schematic illustration of a mature neuromuscular junction (NMJ) and its integrated homeostatic support systems. Central panel shows canonical NMJ ultrastructure comprising a myelinated motor axon terminal containing presynaptic active zones, P/Q-type Cav2.1 calcium channels, synaptic vesicles, and presynaptic mitochondria; a 50–80 nm synaptic cleft containing basal lamina components and collagen-tailed acetylcholinesterase (AChE); and a postsynaptic motor endplate rich in deep junctional folds, crest-localized acetylcholine receptors (AChRs), trough-localized Nav1.4 channels, rapsyn scaffolds, and subsynaptic mitochondria. Perisynaptic Schwann cells encapsulate this synapse for trophic support through GDNF and TGF-β. Top right inset: Canonical agrin-LRP4-MuSK signaling pathway whereby neural agrin (z+ splice form) binds to LRP4 which activates MuSK resulting in a Dok-7–Crk/Crk-L amplification loop driving rapsyn-mediated AChR clustering. Bottom right inset: Protein and organelle quality control involving MuRF1-dependent ubiquitination and p62/SQSTM1-mediated autophagic/lysosomal turnover of AChRs as well as MCU/NCLX-regulated mitochondrial calcium handling and ATP/ROS homeostasis. Bottom panel: Four interdependent functional tiers supporting neuromuscular transmission and myofiber integrity. Created in BioRender. Li, S. (2026) https://BioRender.com/phh7fe9, accessed on 14 September 2026.

Taken together, NMJ homeostasis depends on coordinated postsynaptic signaling, mitochondrial metabolism, autophagy-mitophagy, and Schwann-cell support. These systems do not operate independently. Under pathological stress, they form interconnected feedback loops that determine whether synaptic dysfunction remains reversible or progresses to structural denervation and myofiber atrophy (Figure 1).

## 3. Disease-Specific Molecular Mechanisms of NMJ Degeneration in Different Types of Skeletal Muscle Atrophy

### 3.1. Age-Related Skeletal Muscle Atrophy (Sarcopenia)

Aging is associated with gradual NMJ remodeling, although the process is not uniform across muscles or fiber types. Postsynaptic AChR clusters often shift from continuous pretzel-like structures to fragmented plaques; presynaptic branching may decline, axons may develop bead-like swellings, and the proportion of partially or fully denervated endplates increases [23]. Fast-twitch type IIb/x fibers are generally more vulnerable than slow-twitch fibers, whereas extraocular muscles are relatively resistant [24]. This heterogeneity probably reflects differences in activity patterns, basal MuSK and Dok-7 expression, antioxidant capacity, metabolic demand, and local glial support. The evidence is strongest for age-associated remodeling and functional association; whether NMJ failure is the initial causal event in human sarcopenia remains unresolved.

Mitochondrial dysfunction appears to contribute early to age-related NMJ vulnerability [25]. Reduced PGC-1α activity can suppress the NRF1/2-TFAM axis and impair mitochondrial biogenesis. Lower expression of Mfn1/2 and OPA1, together with increased Drp1-dependent fission, promotes mitochondrial fragmentation. Defective mitophagy permits damaged mitochondria to accumulate at synaptic regions and increase ROS production. These changes could impair vesicle cycling, calcium handling, and postsynaptic signaling stability. Direct evidence that oxidative modification of LRP4 or MuSK is rate-limiting in human sarcopenic NMJ failure remains lacking; current support is stronger for mitochondrial stress and age-related weakening of synaptic maintenance programs than for a specific oxidative lesion in MuSK signaling [25,26,27].

The agrin-MuSK pathway also weakens with age: motor-neuron-derived agrin may decline, LRP4 clustering becomes less stable, MuSK phosphorylation decreases, and Dok-7/rapsyn recruitment is compromised [27]. Neurotrypsin-mediated agrin cleavage increases CAF22, a candidate circulating marker of NMJ remodeling [28]. Schwann cells show senescence-associated changes, including increased p16INK4a and p21Cip1 and reduced GDNF/BDNF production [29]. Increased jitter and conduction block indicate a reduced transmission safety factor and correlate with grip strength or gait speed in some cohorts [30]. Mechanistic priority should therefore be given to interventions capable of improving transmission before changes in myofiber size become evident [31].

### 3.2. Disuse Skeletal Muscle Atrophy

Disuse atrophy differs from denervation. During short-term unloading, the dominant NMJ abnormality is usually functional rather than structural: transmission declines while endplate architecture is often preserved [32,33]. With prolonged inactivity or spinal cord injury, AChR fragmentation, terminal retraction, partial denervation, and endplate remodeling can emerge. This distinction is clinically important because short-term disuse is often reversible, whereas chronic disuse may acquire denervation-like features [34].

Electrophysiologically, short-term disuse can reduce endplate potential amplitude and neuromuscular performance, although presynaptic and postsynaptic contributions vary with model and duration. In longer-term disuse, re-expression of the embryonic AChR γ subunit and increased receptor sensitivity may occur, but this response is less pronounced than the hypersensitivity observed after complete denervation [35]. Reduced mechanical loading downregulates Piezo1-dependent signaling in myofibers [36]. Presynaptic active-zone or vesicle-cycling defects are plausible contributors to fatigue during repetitive stimulation; however, evidence for specific reductions in Cav2.1 density, readily releasable vesicle pool size, or clathrin-mediated endocytosis in disuse remains less direct and is often extrapolated from aging or exercise-active-zone studies [32,33,37].

C-boutons regulate motoneuron excitability through M-current modulation [38]. Aging reduces C-bouton number, whereas short-term disuse does not appear to reduce it further in young or old mice [39]. Older individuals may therefore enter immobilization with a reduced excitability reserve. Because short-term disuse usually preserves basal lamina, produces only modest effects on agrin-MuSK signaling, and retains Schwann-cell support, remobilization can restore NMJ function before chronic remodeling develops [33].

### 3.3. Acute Denervation Skeletal Muscle Atrophy

Denervation remains the clearest setting in which loss of neural input initiates synaptic failure followed by myofiber atrophy. Neural agrin and trophic support are abruptly interrupted; axonal injury initiates Wallerian degeneration and presynaptic calcium disruption; terminal Schwann cells adopt a repair-associated state; and, over days to weeks, presynaptic terminals withdraw, AChR density falls, and endplate architecture fragments [40]. The clinically relevant issue is not only the speed of degeneration but also the narrowing window during which the postsynaptic apparatus remains receptive to reinnervation.

Denervated fibers develop hypersensitivity. Loss of activity induces myogenin, activates AChR subunit transcription, and promotes ectopic receptor expression [41]. The adult ε subunit declines while the embryonic γ subunit reappears, prolonging channel open time. Loss of neural agrin reduces MuSK activation and rapsyn-mediated anchoring, while MuRF1-dependent ubiquitination can accelerate AChR turnover [18]. In chronic denervation, Schwann cells lose regenerative competence and fail to maintain effective Bungner bands, limiting axonal guidance [42]. Repair models further indicate that endplate preservation depends on the guidance scaffold and local repair environment [43].

### 3.4. Muscle Atrophy Associated with Neurodegenerative and Genetic Diseases

CMS and DMD are included not as canonical atrophy syndromes, but as genetically informative comparators that clarify how primary NMJ defects or instability of the postsynaptic architecture can influence muscle wasting and weakness.

#### 3.4.1. Amyotrophic Lateral Sclerosis (ALS)

ALS provides strong evidence for early distal synaptic involvement in genetic and experimental models. In several ALS models, NMJ degeneration precedes overt weakness and motor-neuron soma loss [44]. Direct longitudinal visualization of human NMJs remains limited because ALS is genetically and anatomically heterogeneous. C9orf72 repeat expansion is common in many European-derived populations, whereas variant distributions differ in East Asian cohorts [45]. Dipeptide repeat proteins may impair agrin-LRP4-MuSK signaling and mitochondrial function [46], while TDP-43 pathology can disturb distal axonal RNA handling, local translation, and Rab-dependent vesicle recycling [47]. Muscle-derived extracellular vesicles may further modulate distal axonal vulnerability, although directionality and stage specificity remain unsettled. Autophagy may be protective early and harmful when dysregulated at later stages [48].

#### 3.4.2. Spinal Muscular Atrophy (SMA)

SMA is caused by loss or deficiency of SMN protein due to SMN1 mutation or deletion. NMJ pathology reflects both impaired development and defective maintenance [49]. SMN participates in the assembly and trafficking of RNA granules required for distal axonal protein synthesis. When SMN is deficient, transport of transcripts encoding presynaptic proteins such as Munc13-1 is impaired, active-zone assembly is compromised, Cav2.1 clustering is reduced, and quantal release decreases [50,51]. SMN deficiency can also disturb mitochondrial fusion and axon-muscle communication, potentially weakening trophic support for agrin-MuSK signaling and accelerating synaptic dysfunction [52].

#### 3.4.3. Congenital Myasthenic Syndromes (CMS)

Congenital myasthenic syndromes (CMS) are genetically heterogeneous disorders affecting presynaptic, synaptic cleft, or postsynaptic NMJ components. More than 40 causative genes have been reported [53]. Mutations in AGRN, LRP4, MUSK, DOK7, and RAPSN commonly impair postsynaptic assembly, but treatment responses differ by molecular subtype. DOK7 mutations disrupt MuSK signal amplification and typically produce limb-girdle weakness; β2-adrenergic receptor agonists can improve symptoms, probably through cAMP-PKA-dependent upregulation of AChR, MuSK, and related synaptic genes [54]. By contrast, *COLQ* mutations impair AChE anchoring, causing ACh accumulation, receptor desensitization, and depolarization block. In this subtype, broad activation of MuSK signaling is not an established therapy and could plausibly worsen postsynaptic overactivation. CMS therefore illustrates why NMJ-targeted therapy requires molecular diagnosis rather than pathway-based generalization [55].

#### 3.4.4. Duchenne Muscular Dystrophy (DMD)

In Duchenne muscular dystrophy (DMD), dystrophin at the base of postsynaptic folds links F-actin to the dystrophin-associated glycoprotein complex [56]. Its loss destabilizes junctional folds, fragments AChR clusters, and disperses Nav1.4 channels, thereby lowering the transmission safety factor [57]. Classical work established the subsynaptic cytoskeleton as a determinant of AChR clustering [58]. Emerging evidence suggests that phase-separation-like mechanisms may contribute to postsynaptic organization [59], but this remains an emerging hypothesis until peer-reviewed DMD-specific rescue data are available. The stronger conclusion is that dystrophin loss compromises mature endplate architecture; whether SH3BP2 is therapeutically rate-limiting remains unresolved. It should also be noted that different dystrophin mutations, as well as other genetic myopathies such as dysferlinopathy, can differ substantially in their structural impact and temporal progression, and should not be grouped without mutation-specific evidence [60].

### 3.5. Emerging and Systemic Contexts: NMJ Involvement as a Potential Modifier Rather than a Proven Driver

Before discussing individual systemic contexts, it is important to distinguish shared downstream pathways from disease-specific molecular lesions. Inflammation, oxidative stress, mitochondrial dysfunction, impaired autophagy–mitophagy, disuse, and metabolic stress are common to many atrophy conditions and can influence NMJ stability indirectly [61]. However, their presence does not establish that NMJ degeneration is the primary driver. In contrast, disease-specific lesions directly target defined NMJ components or their upstream regulators. For example, acute denervation interrupts neural agrin and trophic support [40,41,42,43]; ALS involves TDP-43 pathology, C9orf72 dipeptide repeats, and impaired axonal transport [62]; SMA reflects SMN deficiency and disrupted presynaptic active-zone assembly [50]; CMS is caused by mutations in *AGRN*, *LRP4*, *MUSK*, *DOK7*, *RAPSN*, or *COLQ* [63]; DMD involves dystrophin loss at the postsynaptic cytoskeleton [56,57,58]; and chemotherapy can damage mitochondrial DNA and axonal transport [64]. Even within a single gene, different mutations may produce distinct structural and temporal NMJ phenotypes, as seen with different dystrophin mutations and other genetic myopathies such as dysferlinopathy [60]. Distinguishing these categories requires paired assessment of inflammatory and metabolic markers, NMJ morphology, electrophysiology, and longitudinal functional readouts. Moreover, inflammation-driven atrophy and NMJ-dependent atrophy can coexist; for instance, glucocorticoid therapy is a potent anti-inflammatory intervention but can itself induce muscle atrophy independently of NMJ degeneration [65]. Therefore, systemic modifiers should be interpreted as context-dependent contributors rather than as evidence for a universal NMJ-centric mechanism.

#### 3.5.1. Diabetes-Related Skeletal Muscle Atrophy

Diabetes-related NMJ change is best interpreted as a multifactorial modifier arising alongside metabolic stress, vascular injury, and peripheral neuropathy rather than as a primary NMJ disease. Later stages may involve AChR micro-fragmentation, partial denervation, and endplate disruption [66]. AGE-RAGE signaling can alter extracellular matrix stiffness and inflammatory tone [67], while mitochondrial ROS, ischemia, and reduced trophic support may impair repair capacity [68]. Direct evidence that these pathways specifically modify LRP4 or MuSK in human diabetic NMJs remains insufficient. Causal attribution will require studies that measure neuropathy, microvascular injury, neuromuscular transmission, and endplate morphology in the same cohort.

#### 3.5.2. Cancer Cachexia-Induced Muscle Atrophy

Cancer cachexia exposes the NMJ to inflammation, metabolic stress, inactivity, and tumor-derived mediators. Experimental models report denervation-like changes and mitochondrial abnormalities in subsets of endplates [69,70], whereas human rectus abdominis samples from cachectic patients may show preserved NMJ morphology [71]. NMJ pathology may therefore contribute to weakness in selected settings, particularly when inflammation, disuse, and chemotherapy coexist. It should not, however, be described as a dominant cachexia driver without paired activity, treatment-exposure, morphology, and transmission data.

#### 3.5.3. Chemotherapy-Induced Muscle Atrophy

Chemotherapy-related NMJ injury should be distinguished from toxic neuropathy and direct myofiber injury. Platinum agents can damage mitochondrial DNA, impair respiratory-chain transcription, increase ROS, and disrupt calcium homeostasis [72]. Axonal transport impairment and Schwann-cell stress may secondarily reduce terminal support. Increased CAF22 after chemotherapy has been associated with lower grip strength or skeletal muscle index [73], but this association does not prove synapse-specific injury. Pretreatment baselines, neuropathy scales, NfL, CAF22, electrophysiology, and muscle imaging are needed to separate axonal, myofiber, and synaptic toxicity.

#### 3.5.4. COPD-Related Diaphragmatic Muscle Atrophy

**Figure 2:**
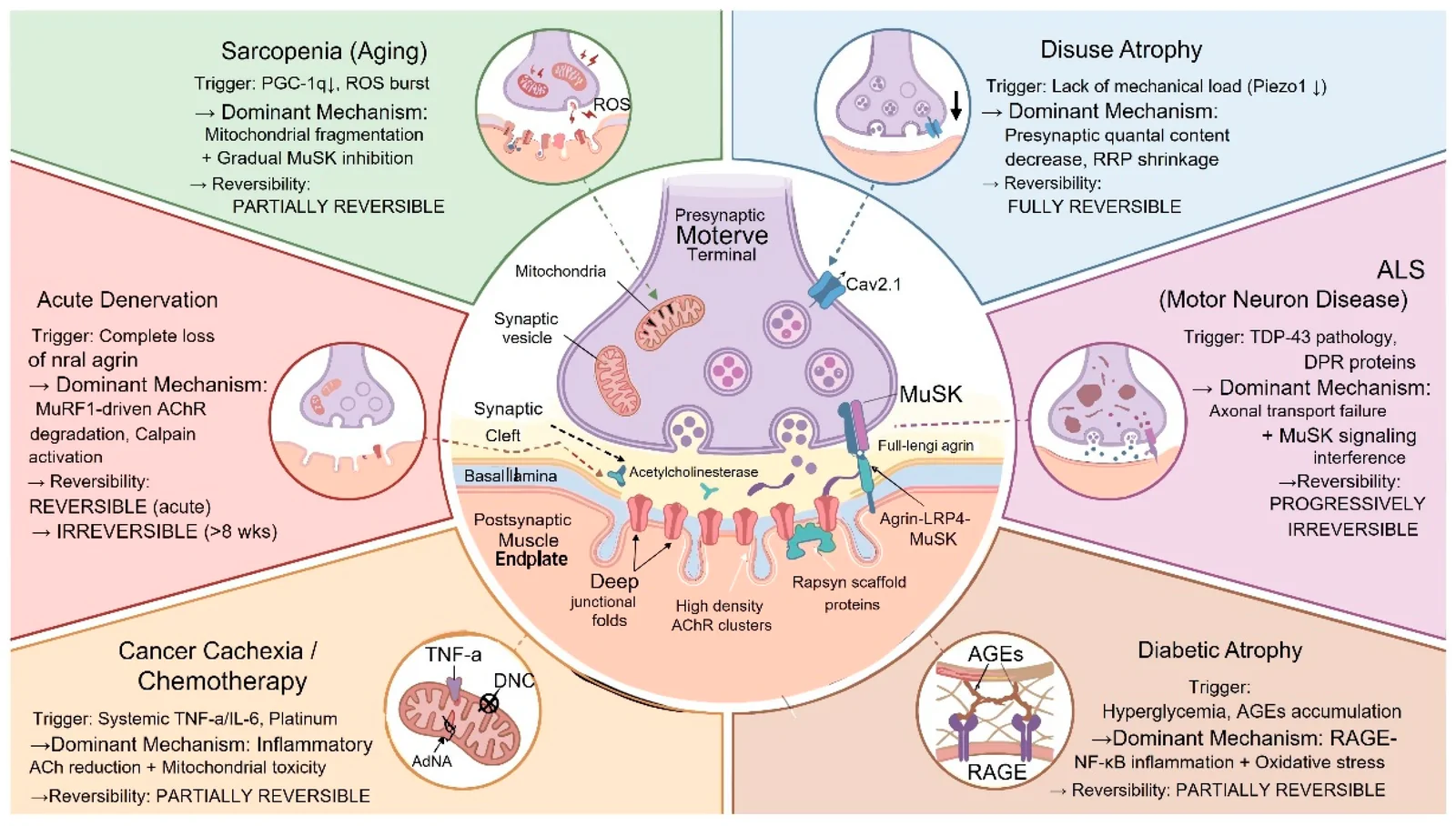
Disease-specific mechanisms, dominant failure modules, and reversibility profiles of NMJ degeneration across muscle atrophy syndromes. Schematic representation centering on normal neuromuscular junction (NMJ) architecture, flanked by six distinct clinical and experimental atrophy contexts. Each pathophenotype is stratified by its initiating trigger, dominant rate-limiting molecular mechanism, and stage-dependent potential for structural or functional reversibility. Disease contexts include age-related sarcopenia, short-term disuse atrophy, acute denervation, amyotrophic lateral sclerosis (ALS/MND), cancer cachexia/chemotherapy-induced wasting, and diabetic muscle atrophy. Created in BioRender. Li, S. (2026) https://BioRender.com/abat10m, accessed on 14 September 2026.

COPD-related diaphragm dysfunction is better framed as respiratory-muscle NMJ vulnerability than as a proven COPD-wide synaptic mechanism. Hyperinflation shortens the diaphragm and imposes mechanical disadvantage, whereas inflammation and hypoxia increase oxidative and proteolytic stress [74]. Experimental smoke-exposure models show diaphragm NMJ fragmentation, reduced terminal coverage, and impaired reinnervation, but direct longitudinal human diaphragm NMJ evidence remains scarce. Limb-muscle findings should not substitute for respiratory NMJ validation (Figure 2).

## 4. NMJ-Targeted Intervention Strategies and Progress in Clinical Translation

### 4.1. Progress in Clinical Research Targeting the Agrin-MuSK Signaling Pathway

The agrin-MuSK pathway is among the best-characterized regulators of NMJ structure and function. By stabilizing postsynaptic AChR clusters and supporting synaptic maturation, it provides a biologically plausible therapeutic target. However, pathway activation should be pursued in a mechanism-specific manner because excessive or inappropriate activation is unlikely to benefit all NMJ disorders.

#### 4.1.1. MuSK Agonist Antibodies

MuSK agonist antibodies bypass upstream agrin-LRP4 limitations and directly promote MuSK activation. ARGX-119 is a humanized MuSK agonist monoclonal antibody with preclinical evidence of NMJ stabilization in DOK7-CMS and other neuromuscular disease models. Human development has reached early-phase testing: a first-in-human study in healthy participants has been registered, and a Phase 1b study in adult DOK7-CMS is listed as an interventional trial assessing safety, tolerability, pharmacokinetics, immunogenicity, and exploratory functional endpoints. Company-reported data are useful for tracking development but should not be treated as peer-reviewed efficacy evidence. Accordingly, ARGX-119 is best described as an early clinical candidate; therapeutic efficacy in CMS or broader NMJ-related atrophy remains unproven until adequately powered, peer-reviewed clinical data are available [75].

#### 4.1.2. β2-Adrenergic Receptor Agonists

β2-adrenergic receptor agonists induce transcription of NMJ-related genes (AChR subunits, MuSK, rapsyn) via a pathway involving cAMP-PKA-CREB; these drugs may be especially useful in CMS where there is an impairment in postsynaptic organization. Clinical trials have shown benefits with salbutamol or similar agents in DOK7-associated CMS, while animal studies demonstrate improved NMJ structure and survival in DOK7-CMS models [76]; their use in sarcopenia has not been well studied. Terbutaline and other agents can increase lean body mass in some healthy volunteers [77] however evidence on improving strength, physical performance, or NMJ function in sarcopenic patients is lacking. Concerns about safety such as cardiovascular effects must be considered carefully in older populations.

#### 4.1.3. Soluble Mini-Agrin and Gene Therapy

Soluble agrin fragments such as NT-1654 supplement functional agrin and activate LRP4-MuSK signaling. NT-1654 improves NMJ structure and function in selected mouse models, including neurotrypsin-driven disassembly and SMA-related pathology [78,79], but completed efficacy-driven human trials have not been established. AAV-DOK7 improves AChR clustering and endurance in animal models [80]. CRISPR-dCas9 activation of endogenous MUSK or DOK7 is conceptually attractive but remains preclinical [81].

### 4.2. Clinical Trial Progress of Mitochondria-Targeted Therapies

Although mitochondrial dysfunction has a role in various types of NMJ degeneration, there are no direct clinical data available. MitoQ decreased oxidative and inflammatory measures in leukocytes from patients with type 2 diabetes; this study did not show NMJ- or sarcopenia-specific efficacy [82]. SIRT1/PGC-1α modulators (SRT2104) were tested for safety and pharmacokinetics in older adults [83]. Claims need translation readouts including validated synaptic markers and function beyond general oxidative stress endpoints.

### 4.3. Clinical Research and Guideline Recommendations for Exercise Intervention

Exercise remains the most consistently supported non-pharmacological intervention for preserving neuromuscular function. Resistance training and combined endurance-resistance programs improve muscle strength and physical performance in older adults and are recommended in sarcopenia management [84]. Mechanistically, exercise can enhance neural drive, maintain AChR clustering, support mitochondrial quality control, and modulate NMJ-related gene expression [85]. Some studies suggest that exercise may reduce circulating CAF22 or other markers associated with NMJ instability, but direct evidence in humans remains less mature than the evidence for global functional benefit. Prehabilitation before unloading can attenuate NMJ remodeling in animal models, although comparable human data remain limited [86].

Exercise should therefore be presented as guideline-supported for sarcopenia, with plausible NMJ benefits rather than as biomarker-directed NMJ therapy. For chemotherapy- or diabetes-related dysfunction, supervised individualized exercise is reasonable, but NMJ-focused endpoints are still needed.

### 4.4. Combination Intervention Strategies

Because NMJ degeneration involves neurons, muscle fibers, Schwann cells, mitochondria, extracellular matrix, and systemic signals, combination therapy may ultimately be required. In animal models, pairing MuSK activation with resistance loading, or combining myofiber anabolic strategies with NMJ stabilization, can produce larger effects than either intervention alone [87,88]. The key principle is to synchronize synaptic protection with myofiber maintenance.

**Figure 3:**
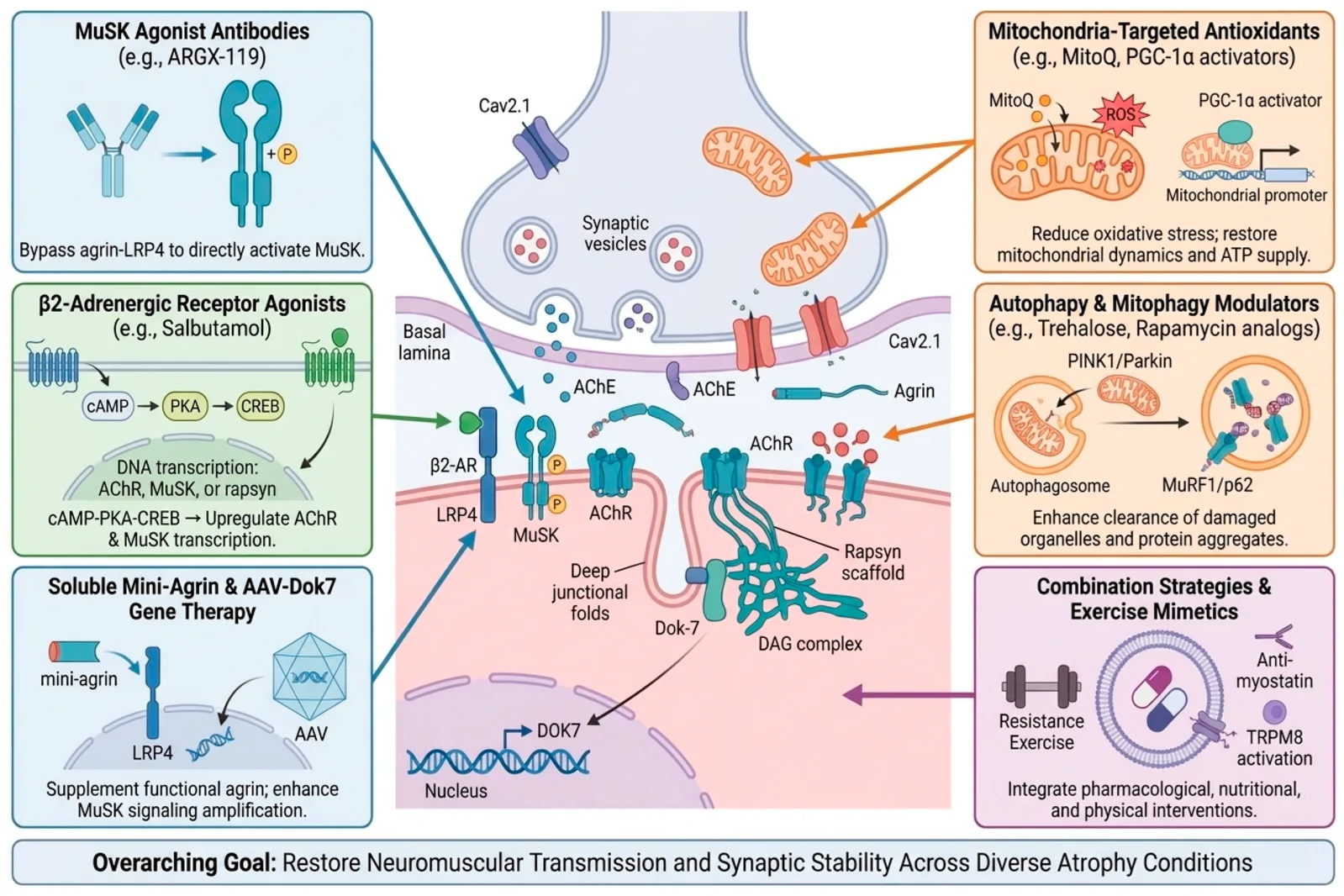
Schematic representation of different therapeutics mapped onto respective molecular and cellular failure modules at the neuromuscular junction (NMJ). Postsynaptic signaling enhancers are highlighted on left panels: MuSK agonist antibodies (e.g., ARGX-119), which bypass agrin-LRP4 requirements by driving receptor auto-phosphorylation; β2-adrenergic receptor agonists that increase AChR, MuSK, and rapsyn transcription through a cascade involving cAMP-PKA-CREB signaling; and soluble mini-agrin or AAV-DOK7 gene therapies restoring signal amplification. Organelle and protein quality control modulators are detailed on right upper panels: Mitochondria-targeted antioxidants (e.g., MitoQ, PGC-1*α*activators) mitigate ROS and boost ATP supply whereas autophagy/mitophagy enhancers clear damaged synaptic components. Integrated combination therapies and exercise mimetics are shown on the right lower panel aiming to synergistically restore transmission safety factor and synaptic stability. Created in BioRender. Li, S. (2026) https://BioRender.com/e138aag, accessed on 14 September 2026.

Combination trials should remain mechanism-led and conservative. Candidate pairings include exercise plus MuSK activation, mitochondrial support plus resistance training, or anti-inflammatory therapy plus NMJ stabilization in cachexia, provided that enrollment, timing, and safety monitoring are biomarker-informed (Figure 3).

## 5. Unresolved Questions, Research Limitations and Clinical Needs

### 5.1. Unresolved Questions in the Field

#### 5.1.1. The Controversy over Causality and Bidirectional Regulation Between NMJ Degeneration and Myofiber Atrophy

NMJ degeneration is often placed upstream of atrophy because impaired transmission reduces activation, promotes proteolysis, and can precede fiber shrinkage. A one-way hierarchy, however, is too simple. The synapse and myofiber influence each other in a disease- and stage-dependent manner.

Muscle-specific MuRF1 deletion in aged mice links myofiber proteolytic pathways to postsynaptic MuSK phosphorylation and AChR fragmentation [18]. Evidence for extracellular-vesicle- or PGC-1α-mediated muscle-to-nerve feedback comes from separate experimental contexts and should be interpreted independently [48]. Thus, NMJ dysfunction may drive fiber atrophy in some settings, whereas stressed myofibers can also feed back onto synaptic maintenance.

**Figure 4:**
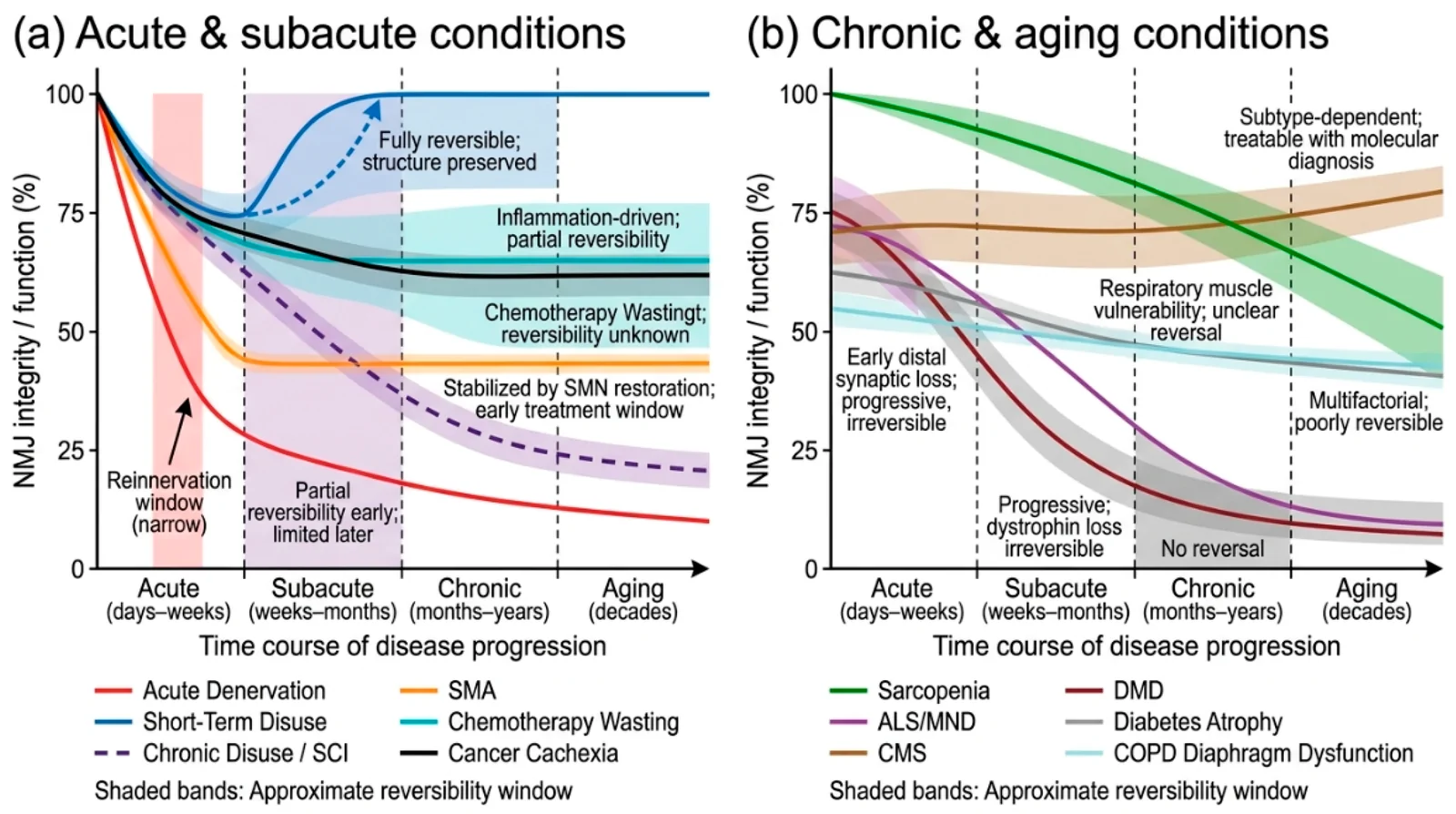
Conceptual time-course of NMJ degeneration across skeletal muscle atrophy conditions. The schematic illustrates the relative progression of NMJ structural and functional integrity over time in different atrophy contexts. The *x*-axis represents the time course divided into acute (days–weeks), subacute (weeks–months), chronic (months–years), and aging (decades) phases. The *y*-axis represents NMJ integrity/function (100% = normal; 0% = complete denervation and irreversible endplate disassembly). Each curve represents a distinct disease or condition, drawn qualitatively based on the evidence discussed in the review. Shaded areas beneath each curve indicate the approximate window of reversibility, which varies from narrow (e.g., acute denervation) to broad (e.g., short-term disuse). Key molecular or therapeutic events are annotated. This figure is intended to summarize temporal logic rather than to provide quantitative data, as the underlying studies differ in species, muscle type, and follow-up duration. Created in BioRender. Li, S. (2026) https://BioRender.com/owtpdmq, accessed on 8 October 2026.

The key question is which direction becomes rate-limiting. In acute denervation, the initiating lesion is neural. In sarcopenia, mitochondrial dysfunction, altered activity, myofiber stress, and glial senescence may progress in parallel. Inducible, cell-type-specific manipulation combined with longitudinal imaging and functional testing is needed to compare synaptic rescue after myofiber injury with myofiber protection after NMJ damage (Figure 4).

#### 5.1.2. The Translational Gap Between Animal Models and Human Disease

Most mechanistic NMJ studies rely on rodent models, yet their predictive value for human disease is limited. This limitation is not merely technical; it reflects differences in disease timescale, muscle composition, NMJ structure, and clinical heterogeneity. For this reason, evidence from rodents should be used to generate testable mechanisms rather than to support unqualified claims about human disease.

Mouse sarcopenia evolves over months, whereas human sarcopenia develops over decades [89]. Mouse limb muscles often contain abundant type IIb fibers, while human muscle is enriched in type I and IIa fibers. Endplate size, AChR organization, activity patterns, and remodeling susceptibility may therefore differ [90]. ALS illustrates a second mismatch: genetically defined mouse models show relatively synchronized pathology, whereas human ALS varies by genotype, muscle region, comorbidity, and progression rate [91].

Two complementary approaches are needed. Improved animal models, including humanized mouse systems and selected non-human primate studies, may better capture aspects of human NMJ biology. At the same time, human iPSC-derived neuromuscular models and organoids offer an opportunity to study patient-specific synaptic phenotypes, drug responses, and genotype-mechanism relationships [92]. These systems are not yet replacements for in vivo models, but they can reduce dependence on rodent biology for target selection and early drug screening.

#### 5.1.3. Why NMJ-Targeted Drug Trials Often Fail to Translate

NMJ-targeted drug development has generated convincing preclinical signals in selected models, but clinical translation has been uneven. Four issues recur: timing, patient selection, delivery, and endpoint choice. Muscle mass, global strength, and physical performance can change for reasons that are not NMJ-specific; without biomarkers or electrophysiology, an apparent clinical response cannot be confidently attributed to synaptic repair.

Clinical trials often enroll patients after weakness is established, when diagnostic delay and structural remodeling may restrict reversibility [93]. Molecular subtyping is also essential: a MuSK agonist may help when inadequate MuSK activation limits AChR stability, but not when the primary defect is AChE anchoring or receptor desensitization, as in COLQ-related CMS [75,78]. Finally, biologics and gene therapies must reach peripheral terminals and endplates at effective local concentrations without excessive off-target exposure [94].

### 5.2. Limitations of Current Evidence

#### 5.2.1. Myofiber Type and Muscle Group Bias in Study Subjects

Current NMJ research is biased toward limb muscles and fast-twitch fibers. Slow-twitch muscles, respiratory muscles, pharyngeal and laryngeal muscles, and extraocular muscles remain underrepresented.

This bias has practical consequences. COPD-related diaphragm dysfunction cannot be fully understood from studies of tibialis anterior or gastrocnemius. Similarly, the relative resistance of extraocular muscles to aging and ALS may reveal protective mechanisms, yet these muscles are rarely used for mechanistic studies. A more clinically relevant research agenda should compare vulnerable and resistant muscles across fiber types, activation patterns, metabolic profiles, and Schwann-cell states.

#### 5.2.2. Insufficient Level of Evidence in Clinical Translational Research

Clinical evidence for NMJ-targeted intervention remains uneven. β2-adrenergic receptor agonists have the strongest support in selected CMS subtypes, particularly DOK7-related disease [95,96]. Many other candidates remain at the preclinical or early-phase clinical stage.

Clinical trials also often lack NMJ-specific enrollment criteria and outcome measures. Patients are commonly grouped by clinical diagnosis rather than synaptic mechanism or biomarker profile. Endpoints such as muscle mass and global strength are important but cannot determine whether a therapy directly improves NMJ integrity. Stronger trial designs would combine clinical function with circulating biomarkers, single-fiber electromyography, repetitive nerve stimulation, motor-unit estimates, and, where feasible, imaging or biopsy-based synaptic endpoints. Combination strategies also remain underexplored despite the multicellular nature of NMJ degeneration.

#### 5.2.3. Technical Bottlenecks in Human NMJ Research

Human NMJ research is constrained by limited tissue access and few tools for longitudinal assessment. Human NMJs are usually studied in biopsy or postmortem samples, which restricts dynamic observation. In vivo two-photon microscopy is valuable in mice but is not clinically scalable because of depth and access limitations. Neuromuscular organoids and microphysiological systems are improving, but many still lack mature Schwann-cell integration, vascular support, adult-like endplate architecture, and long-term stability. Spatial transcriptomic and proteomic maps of human NMJs remain scarce, limiting understanding of human-specific cellular communication at the synapse.

### 5.3. Unmet Clinical Needs and Research Gaps

#### 5.3.1. The Gap in Non-Invasive Early Diagnostic Technology for Human NMJ Degeneration

A major clinical gap is the inability to detect NMJ degeneration before functional impairment develops. Current diagnosis of muscle atrophy usually relies on muscle mass, strength, and physical performance. By the time these measures become abnormal, the therapeutic window for synaptic rescue may already be narrowing. The field needs validated circulating biomarkers, portable electrophysiology, and clinically feasible imaging or surrogate measures that can detect NMJ stress at a subclinical stage.

#### 5.3.2. The Gap in Regulatory Mechanisms of Sexual Dimorphism and Ethnic Differences

Sex differences remain insufficiently studied. Many animal experiments use only male mice, and female participants are underrepresented in some clinical cohorts. Because sarcopenia prevalence and strength decline can differ between women and men [97], the effects of estrogen signaling, mitochondrial regulation, agrin-MuSK activity, and Schwann-cell biology require direct investigation.

Ethnic variation is also underexamined. Populations differ in muscle mass distributions, sarcopenia diagnostic thresholds, ALS incidence, genetic architecture, and environmental exposures [98,99]. At present, there is little evidence to determine whether NMJ degeneration follows distinct molecular patterns across populations. Multicenter cohorts with representative enrollment are needed before biomarker thresholds or therapeutic algorithms can be generalized.

#### 5.3.3. The Gap in Disease-Specific Precision Intervention Strategies

Many proposed NMJ interventions remain broad pathway modulators. Yet the dominant lesion differs by disease: mitochondrial dysfunction and impaired quality control are prominent in sarcopenia; interruption of neural agrin supply defines denervation; systemic inflammation is central to cachexia; and chemotherapy adds mitochondrial and axonal-transport toxicity. Precision therapy will require matching the intervention to the lesion and to the stage at which the lesion remains reversible.

Another unmet need is repair of late-stage NMJ degeneration. Most current strategies aim to preserve synaptic integrity or slow decline. Once complete denervation and endplate remodeling have occurred, protection alone is insufficient. Regenerative strategies must address axonal guidance, Schwann-cell repair capacity, basal lamina remodeling, receptor reclustering, and re-establishment of functional transmission.

#### 5.3.4. The Gap in the Systemic Regulatory Network of NMJ Degeneration

The NMJ also responds to systemic physiology. Circadian disruption, insulin resistance, obesity, chronic inflammation, central motor drive, and the gut microbiota-muscle axis may all influence synaptic maintenance. These topics remain peripheral in NMJ research, but they may help explain why local synaptic interventions fail when systemic stressors remain uncorrected.

## 6. NMJ Degeneration-Related Biomarkers and Clinical Applications

### 6.1. Multicenter Clinical Validation and Clinical Translation Status of Circulating Biomarkers

For clinical stratification, the available tools should be separated by what they measure. CAF22 is a synapse-associated candidate marker of agrin turnover; NfL reflects axonal or motor-neuron injury rather than NMJ-specific damage; SFEMG and repetitive nerve stimulation test the transmission safety factor; MUNE/MUNIX estimate motor-unit reserve; and grip strength or gait speed provide integrated functional outcomes. Combining these classes is more informative than interpreting any single marker as a direct measure of endplate loss (Table 2).

#### 6.1.1. C-Terminal Agrin Fragment 22 (CAF22)

CAF22, generated by neurotrypsin-mediated agrin cleavage, is the most studied circulating marker linked to NMJ remodeling [100]. Clinical studies and meta-analyses report higher CAF22 in sarcopenia and negative associations with grip strength, gait speed, or skeletal muscle index [101]. Exploratory use has also been reported in COPD and treatment-related neuromuscular decline [73,102]. CAF22 is therefore a candidate stratification and monitoring marker, rather than a stand-alone diagnostic test or direct quantitative measure of endplate loss. Assay standardization, reference ranges, disease specificity, renal and inflammatory confounding, and responsiveness to intervention remain unresolved.

In chemotherapy-related dysfunction, increased CAF22 has been associated with lower grip strength and skeletal muscle index [73]. A geriatric metformin study reported improved physical performance with lower CAF22 and NfL, but without structural NMJ endpoints this remains hypothesis-generating for synaptic protection [103]. Stronger claims require parallel morphology or electrophysiology showing that biomarker changes track rescue of neuromuscular transmission.

#### 6.1.2. Neurofilament Light Chain (NfL)

NfL reflects axonal injury and has diagnostic and prognostic value in ALS [104,105]. Outside ALS, NfL can associate with muscle mass and strength, but this association does not make it NMJ-specific [106]. Its main value in this context is to distinguish motor-axon injury from postsynaptic endplate remodeling. Combined NfL and CAF22 profiling may help separate axonal degeneration from agrin-cleavage-associated synaptic destabilization, especially in ALS or chemotherapy-related decline, but remains investigational for sarcopenia, diabetes, and cachexia.

### 6.2. Clinical Application of Electrophysiological Biomarkers

Electrophysiological testing provides functional information that circulating biomarkers cannot. Single-fiber electromyography (SFEMG) detects increased jitter and blocking and remains a sensitive method for assessing impaired neuromuscular transmission, particularly in disorders such as CMS [107]. Repetitive nerve stimulation can reveal decremental compound muscle action potentials when the safety factor is reduced and remains a standard electrodiagnostic tool for disorders of neuromuscular transmission [108]. Motor unit number estimation provides information about functional motor-unit loss and compensatory reinnervation. In aging and sarcopenia, these tools may help identify subclinical neuromuscular dysfunction, but protocols must be standardized for older and medically complex populations [109].

## 7. Research Outlook and Future Priorities

The next phase of the field should move beyond cataloguing additional abnormalities and focus on linking them to timing, reversibility, and patient selection. Atlases and intervention studies will have greater translational value when coupled to transmission, motor-unit integrity, or clinical outcomes.

### 7.1. Human-Relevant NMJ Models and Imaging

Human NMJ research needs tools that answer specific biological questions. Imaging of superficial NMJs would be valuable if it can distinguish endplate fragmentation, partial denervation, and reinnervation over time. Microphysiological models should include motor neurons, mature myofibers, Schwann cells, extracellular matrix, and vascular support, but they must be benchmarked against adult human tissue. Spatial multi-omics will be most informative when paired with electrophysiology, contractile readouts, or clinical phenotypes rather than used only for atlas generation.

### 7.2. Resolving Causality and Systemic Modifiers

Causality between NMJ degeneration and myofiber atrophy is likely to be stage- and disease-specific. Inducible cell-type-specific manipulation, longitudinal imaging, and functional testing can separate synaptic failure from myofiber stress, while sex- and population-balanced cohorts are needed to test whether biomarker thresholds generalize across clinical groups.

### 7.3. Early Diagnostic Frameworks

Early diagnosis will depend on convergent readouts rather than a single biomarker. CAF22 and NfL require assay standardization and reference ranges across age, sex, population background, renal function, cancer status, and neurodegenerative disease. Portable electrophysiology and interpretable predictive models may eventually support an NMJ health index, provided that they are prospectively validated.

### 7.4. Mechanism-Guided Intervention Trials

Mechanism-guided trials need enrollment criteria that match disease mechanism, biomarker profile, and reversibility stage. Delivery remains a major barrier: viral vectors, nanobody-based targeting, and terminal- or postsynaptic-directed systems require rigorous assessment of biodistribution, off-target binding, immune response, and long-term safety.

### 7.5. Clinical Standards for NMJ Assessment

Clinical standards are needed to define when biomarkers, electrophysiology, and imaging can be used in sarcopenia, ALS, CMS, diabetic muscle dysfunction, cachexia, and treatment-related wasting. NMJ dysfunction deserves particular attention when strength loss is disproportionate to muscle mass or appears before obvious atrophy.

## 8. Conclusions

Skeletal muscle atrophy cannot be explained by myofiber size alone. Across aging, disuse, denervation, motor neuron disease, genetic NMJ disorders, systemic inflammation, metabolic disease, and treatment-related wasting, NMJ dysfunction can emerge early enough to affect force generation and, in selected settings, shape subsequent atrophy. The clinically relevant question is when the synapse becomes rate-limiting, which lesion dominates, and whether the lesion remains reversible. Agrin-MuSK failure, mitochondrial dysfunction, defective autophagy-mitophagy, Schwann-cell impairment, inflammatory stress, and axonal-transport defects recur across diseases but with different timing and therapeutic implications. Evidence-informed classification by temporal sequence, dominant mechanism, reversibility, and evidence domain clarifies these distinctions and helps explain why many NMJ-targeted therapies have struggled: trials often occur too late, enroll heterogeneous patients, deliver poorly to the synapse, and lack direct NMJ endpoints. Integration of human-relevant models, longitudinal biomarkers, electrophysiology, and mechanism-guided trials could move NMJ biology from an explanatory feature of wasting to a practical framework for earlier diagnosis, stratification, and therapy.

## Figures and Tables

**Table 1:** Evidence matrix for NMJ involvement across skeletal muscle atrophy contexts.

Disease Context	Animal	Human (Morph/Ephys)	Timing/Reversibility	Biomarkers
Acute denervation (Primary neural trigger)	+++	++ morph; +++ ephys	Early reversible; narrow window	Limited
Congenital myasthenic syndromes (Primary NMJ disease)	++	++ morph; +++ ephys	Variable; subtype-dependent	Genetic diagnosis
ALS/MND (Early distal axonopathy)	+++	+/++ morph; ++ ephys	Early pre-symptomatic; progressive	NfL, CAF22
Spinal muscular atrophy (Developmental & maintenance defect)	+++	+ morph; ++ ephys	Early development; stable after treatment	SMN protein
Age-related sarcopenia (Early modifier; unproven initiator)	++	+/++ morph; + ephys	Chronic; partially reversible with exercise	CAF22 (moderate)
Short-term disuse (Reversible functional lesion)	++	+/– morph; ++ ephys	Rapid onset; fully reversible	Minimal
Chronic disuse/SCI (Delayed remodeling with mixed denervation)	+	+ morph; ++ ephys	Slow progression; limited reversal	Limited
Duchenne muscular dystrophy (Postsynaptic cytoskeletal defect)	++	+ morph; + ephys	Progressive; dystrophin loss irreversible	Dystrophin
Chemotherapy-related wasting (Neuropathy/synaptic toxicity overlap)	+	0 morph; + ephys	Onset with treatment; unknown reversal	++ (CAF22/NfL)
Diabetes-related atrophy (Metabolic/neuropathic modifier)	+	+/− morph; + ephys	Chronic; poorly reversible	+/−
COPD diaphragm dysfunction (Respiratory-muscle vulnerability)	+ (smoke models)	0/+ morph; + ephys	Progressive; unclear reversal	+ (CAF22)
Cancer cachexia (Potential modifier; not proven driver)	+	0/+ morph; 0/+ ephys	Onset with systemic inflammation; unclear reversal	+ (exploratory)

Notes: Evidence scoring: +++ = repeated convergent evidence from independent clinical/preclinical cohorts; ++ = repeated but incomplete/partial; + = limited/indirect; +/− = inconsistent/context-dependent; 0 = absent/not established. Abbreviations: 0/+ = mixed or heterogeneous evidence, ranging from absent/not established to limited/indirect. morph, morphology; ephys, electrophysiology; ALS, amyotrophic lateral sclerosis; MND, motor neuron disease; SMA, spinal muscular atrophy; SCI, spinal cord injury; DMD, Duchenne muscular dystrophy; COPD, chronic obstructive pulmonary disease; CAF22, C-terminal agrin fragment 22; NfL, neurofilament light chain.

**Table 2:** Candidate biomarkers and functional readouts for NMJ assessment.

Marker/Readout	Primary Contexts	Key Utility	Limitations & Current Stage
CAF22	Sarcopenia; chemotherapy decline; COPD	Circulating marker of agrin cleavage; useful for research stratification	Not NMJ-specific; assay standardization pending; affected by age/renal function → Research/cohort
NfL	ALS; chemotherapy neurotoxicity	Strong axonal injury marker; complements CAF22	Not NMJ-specific; elevated in many neurological diseases → Clinical (ALS); investigational for NMJ
SFEMG jitter/blocking	CMS; myasthenic disorders; aging research	Sensitive functional assay of transmission safety factor	Operator-dependent; limited scalability in frail patients → Clinical (CMS); research in atrophy
RNS decrement	CMS; myasthenic disorders	Widely available electrophysiology test	Less sensitive than SFEMG; muscle selection matters → Clinical (CMS); supportive in atrophy
MUNE/MUNIX	ALS; sarcopenia; neuropathy	Estimates motor unit loss and reinnervation	Methodological variability; not purely synaptic → Research & selected clinical use
Grip strength/gait speed	Sarcopenia; cachexia; chronic disease	Clinically meaningful integrated performance outcome	Not mechanism-specific; affected by motivation/comorbidity → Clinical standard outcome
NMJ imaging/biopsy	Human tissue studies; animal models	Direct structural readout of endplate fragmentation/denervation	Invasive or technically immature for routine use → Research tool; future potential

Notes: These tools are complementary rather than interchangeable. Practical stratification should combine a functional readout, a biological marker, and a clinical outcome before patients are assigned to an NMJ-targeted intervention arm.

## Data Availability

Data presented in this study are available on request from the corresponding authors.

## Supplementary Materials

The following supporting information can be downloaded at: https://www.mdpi.com/article/10.3390/ijms27209264/s1, All references cited in Supplementary Table S1 are included in the main reference list (References [1,2,3,4,5,6,7,8,9,10,11,12,13,14,15,16,17,18,19,20,21,22,23,24,25,26,27,28,29,30,31,32,33,34,35,36,37,38,39,40,41,42,43,44,45,46,47,48,49,50,51,52,53,54,55,56,57,58,59,60,61,62,,63,64,65,66,67,68,69,70,71,72,73,74,75,76,77,78,79,80,81,82,83,84,85,86,87,88,89,90,91,92,93,94,95,96,97,98,99,100,101,102,103,104,105,106,107,108,109]).
