# Supplementary material for: Neuromuscular Junction Degeneration in Skeletal Muscle Atrophy: Disease-Specific Mechanisms, Evidence-Informed Stratification, and Therapeutic Windows
Source: Int J Mol Sci. 2026 Oct 19;27(20):9264. doi: 10.3390/ijms27209264 (PMC13705012; doi:10.3390/ijms27209264)
Supplement: Supplementary file 1 [file ijms-27-09264-s001.zip › ijms-4589406-supplementary.pdf]

Supplementary Materials: The following supporting information can be downloaded at https://www.mdpi.com/article/doi/s1

Supplementary Table S1. Representative evidence anchors for the evidence matrix

Disease context / causal status	Representative evidence anchors	Main limitation reflected in rating
Age-related sarcopenia Early modifier; unproven initiator	Aging-model remodeling and exercise-sensitive NMJ morphology [23,24]; mitochondrial and synaptic-maintenance reviews or models[25–27,31]; Schwann-cell aging[29]; electrophysiology/CAF22 literature [30,101,102].	Human longitudinal endplate data remain limited; causality is not established.
Short-term disuse Reversible functional lesion	Unloading/disuse reviews and models [32,33]; hindlimb-unloading axonal excitability [34]; AChR regulation and immobilization pathways [35,36]; active-zone extrapolation treated conservatively [37]; C-bouton/disuse data[38,39].	Human endplate morphology and domain-specific presynaptic mechanisms are incompletely validated.
Chronic disuse / SCI Delayed remodeling with mixed denervation	Disuse and prolonged remodeling literature[32,33,35]; motor-unit excitability and plasticity data [34,39].	Short-term unloading findings cannot be assumed to apply to chronic inactivity or mixed denervation.
Acute denervation Primary neural trigger	Denervation sequence and regeneration framework [40]; denervation hypersensitivity [41]; Schwann-cell senescence in chronic denervation [42]; nerve-repair/endplate preservation data [43]; AChR turnover via MuRF1 [18].	Precise timing windows differ between rodent models and human clinical denervation.
ALS / motor neuron disease Early distal axonopathy	Distal axonopathy and early NMJ involvement [44]; C9orf72 synaptopathy and MuSK agonism in models [45,46]; RNA/protein homeostasis and vesicle pathways [47]; extracellular-vesicle signaling [48]; NfL evidence [105,106].	Direct longitudinal visualization of human NMJs remains scarce; NfL is axonal, not NMJ-specific.
SMA Developmental and maintenance NMJ defect	SMN-deficient NMJ dysfunction and transmission phenotypes [49]; Munc13-1/active-zone mechanisms [50,51]; SMN protein-homeostasis biology [52]; agrin-based rescue in SMA models [79].	Clinical SMN-restoring therapy studies rarely include direct NMJ-specific endpoints.
CMS Primary NMJ disease	Genetic and phenotypic CMS framework [53]; DOK7/COLQ subtype considerations[54,55]; mechanism-based treatment literature[96,97].	Evidence is strong but subtype-dependent; broad pathway activation may not generalize.
DMD Postsynaptic cytoskeletal defect	Dystrophin/DGC role at postsynaptic folds [56,57]; subsynaptic cytoskeleton and AChR clustering[58]; SH3BP2 as emerging preprint hypothesis[59].	Human interventional data showing additive benefit of endplate stabilization are limited.
Diabetes-related atrophy Metabolic/neuropathic modifier	Diabetes-associated endplate changes [67]; AGE-RAGE and extracellular-matrix/inflammatory mechanisms [68]; mitochondrial ROS mechanisms [69].	Difficult to separate NMJ injury from neuropathy, ischemia, and myofiber metabolic damage.

Disease context / causal status	Representative evidence anchors	Main limitation reflected in rating
Cancer cachexia Potential modifier, not proven driver	Perturbed BMP/denervation-like mechanisms in models [70]; mitochondrial dysfunction in cachexia[71]; preserved human NMJ morphology in some cachectic samples [72].	Direct human evidence is mixed and does not establish NMJ pathology as a dominant driver.
Chemotherapy-related wasting Neuropathy/synaptic toxicity overlap	Chemotherapy-induced peripheral neuropathy mechanisms[73]; CAF22/NfL changes during cisplatin-based therapy[74].	Biomarkers overlap with neuropathy and do not prove synapse-specific endplate injury.
COPD diaphragm dysfunction Respiratory-muscle vulnerability	Oxidative stress and diaphragm dysfunction literature [75]; CAF22 COPD cohort [103].	Direct longitudinal human diaphragm NMJ data are sparse; limb-muscle extrapolation is inappropriate.

Notes:This supplementary table anchors the qualitative ratings in Table 1 of the main review. It is not intended to replace systematic evidence grading; rather, it identifies representative studies supporting each disease context and highlights the main limitation that determined conservative scoring.
